# Supplementary figures and images for: Cetylpyridinium chloride and chlorhexidine show antiviral activity against Influenza A virus and Respiratory Syncytial virus in vitro
Source: PLoS One. 2024 Feb 16;19(2):e0297291. doi: 10.1371/journal.pone.0297291 (PMC10871507; doi:10.1371/journal.pone.0297291)

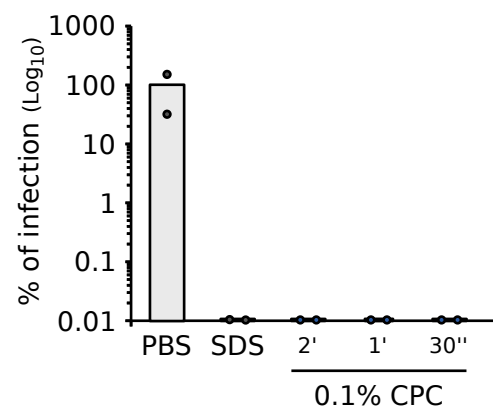

Supplement: S1 Fig — We tested the effect of the exposure time to CPC. To do so, IAV/WSN/33 was incubated with CPC at 0.1% for 2 minutes, 1 minute, or 30 seconds. Next, the virus was diluted and used to infect MDCK cells as previously described. After 48 hours of infection, the viral load was assessed by TCID50. We used a 2-minute treatment with SDS at 0.05% as a positive control and PBS solution as a negative control. No differences in the viral load were observed between the 2 minutes, 1 minute, or 30 seconds exposure. (PDF) [file pone.0297291.s001.pdf]
