# Supplementary material for: Cetylpyridinium chloride and chlorhexidine show antiviral activity against Influenza A virus and Respiratory Syncytial virus in vitro
Source: PLoS One. 2024 Feb 16;19(2):e0297291. doi: 10.1371/journal.pone.0297291 (PMC10871507; doi:10.1371/journal.pone.0297291)
Supplement: Attachment — Submitted filename: Response to Reviewers.pdf [file pone.0297291.s002.pdf]

Response to reviewers

We thank the reviewers for their helpful feedback and for their suggestions to improve our manuscript. We hope we have fulfilled the reviewers' concerns sufficiently. As requested, below you'll find a point-by-point response to each comment. If any additional information is necessary, please do not hesitate to contact us.

Responses are provided in **blue** below each comment. Changes in the Manuscript have been highlighted in **red**.

Reviewer #1

The present in vitro study investigated the antiviral efficacy of cetylpyridinium chloride and chlorhexidine digluconate and various combinations of both for inactivation of Influenza A virus and Respiratory Syncytial virus. The topic of the study is timely, the study has been well-designed and performed diligently. Therefore, there are just some minor points needing revision, as follows:

Title/Abstract:

I would suggest to include the information that the present study is an in vitro investigation in the title and abstract

We have changed the manuscript title as requested.

Introduction:

- ln. 57: please add the following paper as reference for the effects of a combination of CHX and CPC toward SARS-CoV-2: https://pubmed.ncbi.nlm.nih.gov/36942423/
- ln. 61-68: please include references for the statements regarding CHX, e.g. https://pubmed.ncbi.nlm.nih.gov/30967854/

Both references have been included in the manuscript.

Methods:

- I would suggest to include a table with all test compounds, the respective concentrations of antiseptics and the other ingredients (INCI lists)

We have included a table, following a standard INCI List format (Table 1), where all the ingredients of the commercial mouthwash formulations utilized in this work were listed.

Results & Discussion:

- please include the antiseptic concentrations for the commercial products in Figure 1

We have modified Figure 1 following the reviewer recommendation.

- it is a bit confusing to name the two products PerioAid1 and PerioAid2. What about PAAC for Perio Aid Active control and PAIC for Perio Aid Intensive Care?

We thank the reviewer for the suggestion. It is indeed much clearer with the suggested nomenclature. The text and the figures have been modified accordingly.

- please also add information on the ingredients of the mouthwash vehicle (see comment above, suggestion of adding a table)

The requested information has been included in Table 1.

- please include an outlook for future studies, e.g. to conduct a clinical trial and test the effects on intraoral viral load and infectivity (cf.: https://pubmed.ncbi.nlm.nih.gov/35897159/)

We have included these thoughts in the new *Discussion* section.

Reviewer #2

Dear authors,

Your article untitled “Cetylpyridinium chloride and chlorhexidine antiviral activity against IAV and RSV” is an interesting article. However, it should be entirely revised taking into account my comments:

Title

1. Please as your study is based on in vivo studies, you should consider to include “in vitro” in your tittle.

The title has been revised. It now reads: Cetylpyridinium chloride and chlorhexidine show antiviral activity against Influenza A virus and Respiratory Syncytial virus in vitro

Résumé

1. The summary is very short. The material and method should be explained.

2. It could be interesting to structure your abstract: Background, Mat met, Results and then conclusion

Following the reviewer’s recommendation, we have expanded the Abstract section. We have also divided it into *Background, M&M, Results* and, *Conclusion* sub-sections.

Introduction

1. L68 For CHX it will be interesting to precise, as you did for Cetylpyridium, the virus concerned by the antiviral activity. Indeed, CHX acts against CMV, FluV, HBV, SARS-CoV2… (DOI: 10.1177/0022034520967933)

We have extended the introduction section to include the requested information. We have also included new references.

2. L70-73 Could you formulate the aim of your study?
We apologize if the aims of the study were not clear. The corresponding section of the introduction has been rewritten.

Materials and Methods
1. Could you precise for each material (MDCK, DMEM…) the manufacturer, the city and the country?
The missing information has been included.

2. Why did you dilute CPC in PBS and CHX in water? Why did not use the same solution: water or PBS?
3. Why did you do different dilutions for CPC and CHX?
4. L85 to 94 Please can you give more explanations? The steps are not clear for me. You write "the solutions" (line 88): What are these solutions?
We deeply apologize for the confusing explanation. We have rewritten the entire paragraph and hope it is now clear; It was not our intention to mislead the reader.

5. How did you choose the commercial mouthwashes? Did you test all the mouthwashes commercially available and containing CPC ou CHX?
As stated in the manuscript DENTAID partially funded this study and supplied the mouthwashes. We believe the commercial formulations used in our work provide a solid indication of what we can expect from other mouthwashes with similar CPC and CHX concentrations.

Results and discussion
1. This section should be entirely revised because there is only results and no discussion. Discussion comparing the results of this study with previous ones is essential.
2. Please add in the discussion section the limitations of this study
3. Please discuss your choice of analyzing only CPC and CHX because other products could be interesting (DOI: 10.1177/0022034520967933)
These and other thoughts are now discussed in the newly added Discussion section.

References

1. It is important to include more references to discuss your results

As a result of extending the introduction and particularly the discussion section, we have included new references.

1. Line 178 Replace “Acknoledments” by “Acknowledgments”

We thank the reviewer for identifying this typo, it has been corrected.

Reviewer #3

Dear authors,

Thank you for the opportunity to review the interesting manuscript dealing with a clinically relevant topic. However, there are some major point contradicting publication, as listed below:

Title:

From the title, the reader gets no information about the kind of study (is it a clinical study, is it an in vitro study etc.). Please adapt.

We have changed the title to indicate the type of study conducted.

Abstract:

The abstract does neither contain information about the methods used to obtain the findings, nor the statsitical methods, nor about numercial data. Basically, the reader gets no information about the study at all from the abstract.

We have rewritten the abstract. It is now divided into *Background*, *M&M*, *Results* and, *Conclusion* sub-sections. We hope the new wording and structure provide enough information about the content of the manuscript.

The following sentence is unclear and not precise, please provide more information: "Not only do new viruses enter the playground but the impact of old ones is also augmented due to climate change(4)."

We wanted to indicate that not only new respiratory viruses are emerging but also that the impact of current circulating viruses is increasing due to climate change. We have rewritten the sentence. We hope it is clearer now.

There is another clinical study with CHX/CPC combination against SARS-CoV2. Please add https://pubmed.ncbi.nlm.nih.gov/36942423/

As suggested by the reviewer we have included the missing reference.

Regarding the mechanisms, you mostly described antibacterial effects, but almost no antiviral mechanisms. As viruses are within the scope of your study, you should add these informations.
When describing the mechanism of action we referred to “biological membranes” which include bacterial membranes but also the viral membrane in the case of enveloped viruses. We modify the text to clarify this concept.

Please use a clearer (null)-hypothesis for your study.
We have included in Figure legend 2 the null hypothesis of our statistical analysis: “H_0: μ=average viral titer of PBS treated samples.”

Mat/Met:
Please provide a definition for each abbreviation with the first appearance (e.g. MDCK).
We apologize for the missing information. We have included the definition for each of the abbreviations.

Why did you use different concentrations for CHX and CPC? This should be explained in the Mat/Met or the discussion part.
The range of concentrations for CPC and CHX was selected based on the product information of the main over-the-counter mouthwashes according to their active ingredients list. This explanation has been included in the *Discussion* section.

It is not clear, if you used experimental or commercially available solutions of CHX or CPC. Please rewrite the corresponding part.
The CPC, CHX, and mouthwash solutions were provided by DENTAID. This information has been included in the Material and Methods.

Please explain the RSV (with abbreviation definition as well) production in more detail.
We have incorporated a section that indicates how the RSV-mKate2 was generated. Furthermore, we have extended the information regarding the RSV viability assays.

Please add a part regarding the ethical disclosure for using cells and a paragraph explaining the statistical methods.

As far as we are concerned PLoS ONE does not requires an ethical statement for the use of cell cultures. Nonetheless, we have informed the editorial team of the reviewer's concern and provided them with an ethical statement in case they choose to include it.
We have included in the M&M a section regarding the *Statistical analysis*. Note that we have kept some of this information in the Figure 2 legend.

Results/Discussion:

These parts should be seperated according to the submission guidelines. Furthermore, some informations are missing:

-Which statistical tests are the base of the asterisks and statistcial differences?

-What probable explanation do you have for the different performances of CPC and CHX and whiy did you use different concentrations from both substances?

-Please add a paragraph diuscussing the limitations of the in vitro design, especially regarding differences to the oral cavity.

As requested we have separated the *Results* and *Discussion* sections. We apologize for the condensed version of the original manuscript. All the requested information is included in the new *Discussion* section.
