# Supplementary material for: Cetylpyridinium chloride and chlorhexidine show antiviral activity against Influenza A virus and Respiratory Syncytial virus in vitro
Source: PLoS One. 2024 Feb 16;19(2):e0297291. doi: 10.1371/journal.pone.0297291 (PMC10871507; doi:10.1371/journal.pone.0297291)
Supplement: Attachment — Submitted filename: Response to reviewers.pdf [file pone.0297291.s003.pdf]

PONE-D-23-32284R1

Reviewers' comments

We thank the reviewers for their compliments. We hope we have fulfilled the reviewers' concerns sufficiently. As requested, below you'll find a point-by-point response to each comment. If any additional information is necessary, please do not hesitate to contact us.

Responses are provided in **blue** below each comment. Changes in the Manuscript have been highlighted in **red**.

Reviewer #1: All comments raised by this reviewer have been addressed sufficiently. Congrats to a very nice paper!
Thank you very much. We truly believe the reviewers have improve the quality of the manuscript.

Reviewer #3: Dear authors,
Thank you for the renewed opportunity to review the manuscript.
Although improved in some points, there are still major points that need adaptation, especially in the newly written parts.

Thank you very much for your time and dedication to improving the quality of our manuscript.

Abstract:
The results part, although in better structure now, does still not provide numerical data. Please add.
We have incorporated the reduction values associated with each of the tested treatments for both RSV and IAV.
Typo: "CPD" in the conclusion of the abstract.
Thanks for identifying this typo. We have fixed this error and checked the entire document for additional typos.

Introduction:
Please be more concise regarding the mechanism of climate change leading to more viruses.
We have included more information regarding the relation between climate change and the increase in respiratory infections. However, we have kept it brief since this is but a motivation to focus on respiratory viruses.

Mat/Met:
As "pfu" is not an SI-unit, you should define it with the first appearance.
The pfu is defined in the second paragraph of the Materials and Methods which corresponds to its first appearance.
The bracket containing DMEM and FBS is unclear.
We have changed our phrasing, we hope the new wording is clear.
As for all other companies, the location and state of Dentaid should be named with the first appearance.
We have included the missing information the first time Dentaid is mentioned.
This sentence is extremely complicated and hard to understand and should therefore be simplified and shortened: "To generate this virus the RSV infectious clone system, obtained from BEI Resources (NIAID, NIH: Bacterial artificial chromosome plasmid pSynkRSV-I19F containing antigenomic cDNA from RSV A2-Line19F, NR-3646), was transfected into HEK-293 cells together with a codon-optimized T7 polymerase (Addgene, United States, 65974), and an RSV N, P, M2-1, and L expression plasmids at a ratio of 4:2:2:2:1."
We have changed the sentence. We hope it is clear now.

Discussion:
L.287: How do you define a "large study"? Please specify.
By “large” we meant the most comprehensive study. We have changed the text accordingly.
Some of the new paragraphs in the discussion are lacking relevant references, especially 295-308. Please add.
We apologize if the statements in the discussion seemed unsupported by the literature. We have added references in those places in which we felt they were necessary.
